# Supplementary material for: Close-kin mark-recapture methods to estimate demographic parameters of mosquitoes
Source: PLoS Comput Biol. 2022 Dec 12;18(12):e1010755. doi: 10.1371/journal.pcbi.1010755 (PMC9779664; doi:10.1371/journal.pcbi.1010755)
Supplement: Attachment — Submitted filename: sharma2022.pdf [file pcbi.1010755.s002.pdf]

1. Close-kin mark-recapture methods to estimate demographic parameters of mosquitoes

The paper by Sharma et al. presents a Monte Carlo analysis of the use of close-kin mark-recapture (MR) methods for estimating demographic parameters of mosquitoes: here, *Aedes aegypti* is used as the particular species case study. In this approach to MR experiments, genetic markers allow pairs of individuals of different kinships to be identified which informs about the underlying population dynamics of mosquito populations. The authors derive a number of kinship probabilities for pairs of mosquitoes collected at two sampling time points assuming a discrete-time model of mosquito population dynamics. These probabilities include mother-offspring, father-offspring, full siblings and half siblings: in each case across a range of life stages encompassing pupa, larva and adults. Using these results, they then derive pseudo-likelihoods which allow estimation of the underlying demographic parameters from mosquito collection data. Using a stochastic model of mosquito population dynamics of the same form as the generative model used to derive their probabilities and likelihoods, they then generate *in silico* mosquito collection data. These data are then used in a type of Monte Carlo analysis, where the authors probe the use of their methods for estimating key demographic parameters such as the adult population size and the mortality rate.

I enjoyed learning about this approach to estimating mosquito demographic parameters, particularly because I was completely naive to it beforehand. I also commend the authors for their approach, since evaluations of statistical methods on simulated data are unfortunately overly rare. Knowing demographic parameters of mosquitoes is extremely valuable and current methods are weak, so I can really see the importance of an approach such as this. Because of the potential utility of this method, though, I do feel that there are a number of additional analyses which, if done, would boost the paper. Additionally, I also think that the paper narrative and form could do with a fair amount of work, much to condense and focus the prose. Should these be done, I would be happy to recommend this piece for publication in this journal. Yours, Ben Lambert

1.1. Substantive

- I found the manuscript thorough, but I did find reading it felt a bit like, "a collection of things: one after the other", and I think that it could benefit from a substantial condensing which might help its clarity of message. For example, it is commendable that the authors go through so much effort to explain their calculations, but the flipside of this is that Section 2.2 is mammoth (pages 4-13!), currently, and a bit of an effort to get through. I think that, since much of the detail in the calculations is similar across the different kinships considered, the manuscript would benefit greatly from a rejigging: explaining one / two max calculations in detail, then tabularising the rest / putting them in supplementary information. The same logic applies to the likelihoods section (pages 13-16), which could be condensed to aid readability. Additionally, I feel like the expressions for the various kinship probabilities are

full of intuition (and, to be fair, the authors do go into detail to explain their derivation), and that this could nicely be illustrated by some plots. For instance, I would find it informative to see how p_MOA depends on a) the difference between t_1 and t_2; b) (say) the lifespan of adults. I feel like some graphical probing of these quantities would demystify how the method works.

- In similar vein to above, I do wonder whether there is a more interesting way to probe the method and illustrate how it performs: all the results figures are box plots of estimates vs actual across various parameter sets. One thing I wondered was, would the estimation method you employ be able to detect changes in the mosquito mortality / population size as the result of an intervention (e.g. IRS / bednet distribution, larviciding etc.)? If so, how big a shift would be required for this to be possible (i.e. could you do a power analysis)? This could be important if deciding to use such an approach as part of an RCT to evaluate a new product, and I think, could lead to a nice figure.
- "...three months, which is consistent with the length of a season, during which time the constant population size assumption in this analysis approximately holds". I think it's fine to start off, as you do here, by assuming a population at equilbrium, but I think it might be worthwhile considering how robust your inferences would be should this not be the case. One of your results which I suspect would be affected quite strongly by non-equilibrium populations is Figure 4C: which shows that sampling frequency has only minor effects on the estimates. As such, I wonder whether, "The key result from this analysis is that CKMR estimates of NA and μA are robust for daily, biweekly, weekly, and even fortnightly collections, which is reassuring for the logistical feasibility of the method" might be a little strong given likely variability in emergence in the field. I feel like a simulation analysis, where you allow emergence rates to vary and see when this breaks your model assumptions, would be really useful. But this may be OTT for the current manuscript, and I will leave it to the editor to determine whether they think this is in remit.
- In similar nature to the above, did you do any simulations when you examined how sensitive your estimates were to the number of mosquitoes found each day? I.e. if, due to clumping of captures, you captured 20 mosquitoes on some days, and zero on others. It wasn't clear to me whether you allowed stochasticity in the numbers captured each day, but I may have missed this.
- "For CKMR methods, temporal information is of utmost importance, and so the day of collection should be known." As you mention, in typical surveillance programmes, this is not the case. But would you still be able to determine useful estimates should say the day of sampling be known to within 2, 3, 4, etc days? Of course, this would mean that you'd have another variable that you would need to marginalise over, but I'm not convinced it would necessarily break your analysis entirely and could substantially curb costs.
- I think it's great that the software for this paper is made openly available via: https://github.com/GilChrist19/mPlex. I did think that the CKMR repo homepage could do with a little more information on how to get the package installed, however; it would also be good to know what dependencies are required to run the simulations. Additionally, if at all possible, it would be good to have the simulations run for the manuscript included within (say) a Makefile (perhaps in a separate repository?), so that someone wanting to recapitulate the analyses would be able to straightforwardly do so. I think that something like this would be extremely helpful given the combinatorics of the experiments you perform (e.g. the variety of parameter sets convered in Figure 4), but I leave it up to the authors / editor to decide where to draw the line. Similarly, have the authors considered tagging a release of code (on

Github) which was used to produce this analysis? This would be handy, especially if, in the future the codebase is modified, so that someone wanting to exactly reproduce this study could access the legacy code through the release.
- Is the random sampling assumption mentioned somewhere? I.e. that individuals captured in the wild represent random samples from the underlying population. I don't have as much experience with *Aedes* as I do with *Anopheles*, but I feel like this assumption might be quite crucial and could lead to biases in estimates (e.g. if host-seeking females which correlates with their age are more likely to be caught in traps). I feel like this could be touched upon in the discussion, particularly because, for *Anopheles* (which is proposed as an additional genus on which to use this method), it is likely an issue.

1.2. Minor

- Throughout the manuscript, the word "consistent" appears (49 times!). For instance, "considering all consistent event histories", "...for consistency with the larva being present on the day of sampling...", "Adult offspring at time t2 from all adult females at consistent times". I think in quite a few of the language could be simplified, often because the quantities are at equilibrium, so consistency isn't really needed. I also didn't necessarily find the use of "consistent" to be that illuminating: e.g. "is summed over days (0 − TE − TL − TP − TA) through (0 − TE − TL − TP), for consistency with the adult offspring being present on the day of sampling " could be changed to "is summed over ... through ... to allow for the range of conceivable times when the adult sampled on day 0 was born as an egg."
- I understand eq. (8) and why p_a(t) -- the probability that a given adult in the population has age t -- enters the equation in the way it does. But it took me a bit of time to understand it. I think it would be good to give a little more explanation of this. I think part of the confusion is the sentence, "pA(t1 − ti) represents the probability that the mating event occurs on day (t1 − ti)." -- it does, but really this just represents the probability that a mosquito is of age (t1-ti).
- I agree with assuming that the adult (and other life stages') mortality rate is independent of age, but I think it's worth saying this somewhere.
- The panels in Figure 2 are not referred to in order: panel B is referred to first, for instance. I think it would be nice if the panels followed the order of the text, since the reader then doesn't find themselves thinking, "Did I miss something?"
- Why not put a legend with "circle -> living individuals; square -> sampled individuals" in figure 2 somewhere as there's quite a bit of space. Also, what do the colours mean? E.g. grey in panel E.
- "For 193 convenience, let us refer to the larva sampled on day t1 as individual 1" -- how about "cohort 1" -- I found the "individual" in this confusing since there are many.
- "pL(t1 − y1 − TE) represents the probability that egg 1 is laid on day (t1 − y1 − TE)". I think it would be more useful to introduce what p_L(t) is (i.e. the probability that a given larva has a given age) first. Then pL(t1 − y1 − TE) is just the probability that a larva is of age (t1 − y1 − TE).
- "the size of each sampling event is sufficiently low relative to the total population size" -> "each sample taken is small relative to the overall population"
- "The kinship probabilities for each pair of individuals are assumed to be independent of each other, even though they are not." I need a little hand-holding here: can you perhaps add a sentence or so that explains this in more detail? Is this just saying that, by removing a small number of individuals from the population, when sampling, does not change the underlying distributions by much? So, for example, in equation (22) you assume that finding

one individual with a given mother does not affect your probability of finding another. In other words, you assume that you sample with replacement whereas, in reality, this is not the case.

- I feel that writing equation (22) (and other similar equations, e.g. eq. (25)) as: k ~ binomial(n_A, p_MOA) or binomial(k|N_A, p_MOA) rather than writing out the explicit binomial likelihood would be more illuminative and also condense things. Then equation (23) could be written as a sum over binomial_log(k|N_A, p_MOA) which would mean the reader has to do less work to follow subscripts and indices.
- "Additionally, when counting siblings, we only consider siblings with indices > i to avoid double-counting." I am not sure where this enters equation (25) or (26)?
- "Functionality is included to account for spatial population structure, with mosquitoes being distributed across populations in a metapopulation [26], and each population having an equilibrium adult population size, N_* A, and exchanging migrants with the other populations; however, in the present analysis, a single panmictic population is modeled." Given that spatial modelling is not included in this analysis, I am not sure it needs to be mentioned as an inattentive reader might start thinking that the model used is spatial?
- "Daily mortality rates are as defined earlier and implemented according to a Bernoulli distribution for each individual." -> "Individual survival through a given day is assumed to follow a Bernoulli distribution with a stage-appropriate survival probability."
- "Sex is modeled at the adult stage - half of pupae emerge as females, and the other half as males, implemented according to a Bernoulli distribution with probability 0.5" -> "The sex of an emergent pupa is drawn from a Bernoulli distribution with probability 0.5, so that, on average, half adults are female and half male."
- "logistically-feasible" -> "logistically feasible" since words ending in "ly" typically don't need hyphens
- "by requiring integration over six latent event times" - I know what you mean but, since your analysis is all discrete, you might want to change "integration" to "summing"?
- "To estimate adult parameters, our default sampling scheme consisted of a total of 1,000 sequenced individuals sampled daily..." -- sounds like a lot of mosquitoes until I read the bit in parentheses. It might sound less alarming if you said, "To estimate adult parameters, our default sampling scheme consisted of a 3 month window when, overall, 1000 mosquitoes were sampled, with roughly uniform numbers of mosquitoes sampled each day (i.e. 11-12 mosquitoes per day)."
- "limited to scenarios where the number of sampled adult females was greater than or equal to the number of sampled adult males" -- I spent a while looking for this ratio until I interpreted what your labels meant in Fig. 4A (e.g. 50F25M25L). I feel that putting a short sentence to explain one / a couple of these would help the slow reader like myself.
- Out of interest, what do you think leads to the bias seen in Figures E and F for short durations of sampling?
- "This confirms that the parameter estimates from the optimal sampling scheme in Figure 4 are indeed optimal..." -- I'm not sure I understand why this follows from the previous sentence.
- "Curiously, we found that including half-siblings in our analyses introduced significant biases in our parameter estimates, leading to an underestimate of μA and an overestimate of NA" I also found this quite strange and I wonder whether there are any ways to probe this further?
- " spatially-structured" -> " spatially structured"
