# Supplementary material for: Close-kin mark-recapture methods to estimate demographic parameters of mosquitoes
Source: PLoS Comput Biol. 2022 Dec 12;18(12):e1010755. doi: 10.1371/journal.pcbi.1010755 (PMC9779664; doi:10.1371/journal.pcbi.1010755)
Supplement: Attachment — Submitted filename: SharmaEtAl_2022_R1_ResponseToReviwers.pdf [file pcbi.1010755.s003.pdf]

Berkeley Public Health

UNIVERSITY OF CALIFORNIA, BERKELEY

DIVISIONS OF BIOSTATISTICS & EPIDEMIOLOGY
SCHOOL OF PUBLIC HEALTH
2121 BERKELEY WAY #5302
BERKELEY, CA 94720

16 August 2022

To: Drs. Penny Hancock and Ville Mustonen, PLoS Computational Biology

Re: PCOMPBIOL-D-22-00257

Please find enclosed our revised manuscript entitled, "Close-kin mark-recapture methods to estimate demographic parameters of mosquitoes" for consideration as a research article in PLoS Computational Biology. We greatly appreciate the round of reviewers' comments and believe we now have a greatly refined manuscript thanks to their thoughtful comments. We have constructively addressed each point below, with reviewer comments in black text, our response in blue text, and quotations from the revised manuscript in blue italics text and quotation marks. We thank you and the two reviewers very much for your time and for improving the quality of our manuscript. We look forward to hearing your response regarding the revised manuscript.

Yours sincerely,

Dr John Marshall, on behalf of the authors

Associate Professor in Residence
Divisions of Epidemiology & Biostatistics
School of Public Health
University of California, Berkeley
Phone: +1-510-664-4724
Email: john.marshall@berkeley.edu
Website: www.MarshallLab.com

Reviewer comments:

****

Editors:

Comments:

Both reviewers recognise the value of this work, which addresses an important question relating to the paucity of demographic data describing mosquito populations, and uses an interesting computational approach to estimate demographic rates from close kin mark recapture experiments. Reviewer 1 recommends some substantial changes and reviewer 2 raises some important caveats to the analysis that need to be emphasised in the written text of the manuscript.

We thank the reviewers and editors for their thoughtful and constructive comments. We have addressed the substantial changes and caveats where we see fit, as described in detail below.

I agree with Reviewer 1 that the readability of the methods section would benefit from a rewrite to capture the equations conceptually in the main text (perhaps some illustrative plots would be helpful) and designate repetitive material to an appendix.

As detailed below, we have: i) edited the Methods section to better characterize a smaller number of equations, ii) designated additional equations to the SI, iii) adapted Figures 2 and 3 so that they better capture the essence of the kinship probability equations, and iv) added Figure 4 to illustrate the temporal nature of the close-kin equations.

I also agree that the performance of the method when the population is not in equilibrium (growing, declining or fluctuating seasonally) needs to be clarified in order to provide a balanced assessment, and I'd be particularly interested to know how this influences estimation of daily larval mortality.

We agree that the performance of the method during periods of population growth and decline and seasonal fluctuations is important to characterize; but consider this to be beyond the scope of the current submission. We intend for this to be the first of a handful of papers exploring the application of CKMR methods to mosquito species, and believe this paper in its present form to be a significant contribution to the field. Of note for subsequent papers applying these methods to mosquitoes, a paper applying CKMR methods to fish did estimate a population growth rate alongside other demographic parameters (Hillary *et al.*, 2018), and subsequent mosquito CKMR work could explore periods of population growth and decline separately, provided this allows for sufficient sampling duration. Alternatively, estimating parameters in the face of seasonal fluctuations would also be interesting. Although not quite the same thing, we note that the mosquito populations that we simulate do in fact fluctuate, both due to stochasticity, and due to lethal sampling. Particularly when sampling is less frequent (e.g., fortnightly), relatively large lethal samples are taken every two weeks, leading to regular population suppression and rebound to which parameter estimation seems to be robust. Nevertheless, we have noted this as an important area for further investigation and inclusion in subsequent papers.

Reviewer #1:

Comments:

I enjoyed learning about this approach to estimating mosquito demographic parameters, particularly because I was completely naive to it beforehand. I also commend the authors for their approach, since evaluations of statistical methods on simulated data are unfortunately overly rare. Knowing demographic parameters of mosquitoes is extremely valuable and current methods are weak, so I can really see the importance of an approach such as this. Because of the potential utility of this method, though, I do feel that there are a number of additional analyses which, if done, would boost the paper. Additionally, I also think that the paper narrative and form could do with a fair amount of work, much to condense and focus the prose. Should these be done, I would be happy to recommend this piece for publication in this journal.

We thank the reviewer for their thorough reading of the paper and constructive comments. We also thank the reviewer for their suggestions regarding additional analyses. Several of these have been added to the revised version of this paper, while there are others that we reserve for subsequent papers. We see this paper as the first in a series that will address the application of these methods to mosquitoes. We appreciate the suggestion to condense and focus the prose, and to refine the paper narrative. We have condensed the paper where possible, moving additional content to the SI. We have also included revised and additional figures to better explain the workings of CKMR methods applied to mosquitoes. We describe these modifications further below.

Substantive comments:

I found the manuscript thorough, but I did find reading it felt a bit like, "a collection of things: one after the other", and I think that it could benefit from a substantial condensing which might help its clarity of message. For example, it is commendable that the authors go through so much effort to explain their calculations, but the flipside of this is that Section 2.2 is mammoth (pages 4-13!), currently, and a bit of an effort to get through. I think that, since much of the detail in the calculations is similar across the different kinships considered, the manuscript would benefit greatly from a rejigging: explaining one / two max calculations in detail, then tabularising the rest / putting them in supplementary information. The same logic applies to the likelihoods section (pages 13-16), which could be condensed to aid readability. Additionally, I feel like the expressions for the various kinship probabilities are full of intuition (and, to be fair, the authors do go into detail to explain their derivation), and that this could nicely be illustrated by some plots. For instance, I would find it informative to see how p_MOA depends on a) the difference between t_1 and t_2; b) (say) the lifespan of adults. I feel like some graphical probing of these quantities would demystify how the method works.

Reviewer 1 is correct that much of the kinship probabilities and likelihoods are similar; however Reviewer 2 suggests that the level of detail is justified. We concluded that some shortening of the Methods section could be achieved; but that reducing the number of equations down to 1-2 would be too much. It is worth noting that the suggestion of Reviewer 1 to put most of the equations in the SI was done in the original submission, with ~100 equations already included in the S1 Text. Towards this goal, we now include what we consider to be a bare minimum of equations in the manuscript: i) mother-larval offspring, ii) father-adult offspring, iii) full-siblings (larva-larva), and iv) half-siblings (larva-larva). The remaining equations are included in the SI. Each of the mother-offspring, father-offspring, full-sibling and half-sibling cases have unique considerations, and at least one comparison involving distinct sampled life stages must be made. By focusing on larvae in all except the father-adult offspring case, the equations are reported in their simplest form, with the more detailed pupal and adult-stage equations being included in the SI. The number of cases described in the text has therefore been reduced from six to four - something we consider to be a reasonable compromise between the suggestions of the two reviewers.

Additionally, in response to Reviewer 1’s suggestion, the revised Figures 2 and 3 have been updated to better explain the kinship probability equations. These figures now walk the reader through each step of the equations by annotating the life history diagrams. We have also added a new figure - Figure 4 - providing a graphical illustration of how selected kinship probabilities depend on the difference between t_1 and t_2.

The pseudo-likelihood equations for parent-offspring and sibling cases have been retained in the manuscript because each takes quite a different approach to pseudo-likelihood calculation. That said; the pseudo-likelihood equations have been simplified in two ways: i) they have been simplified in form, describing distributions wherever possible rather than equations, and ii) the binomial equations for full-siblings have been absorbed into the multinomial equations for full and half-siblings, reducing the number of pseudo-likelihood equations by a third.

In similar vein to above, I do wonder whether there is a more interesting way to probe the method and illustrate how it performs: all the results figures are box plots of estimates vs actual across various parameter sets. One thing I wondered was, would the estimation method you employ be able to detect changes in the mosquito mortality / population size as the result of an intervention (e.g. IRS / bednet distribution, larviciding etc.)? If so, how big a shift would be required for this to be possible (i.e. could you do a power analysis)? This could be important if deciding to use such an approach as part of an RCT to evaluate a new product, and I think, could lead to a nice figure.

We thank the reviewer for their suggestion. The ability of this method to detect intervention impact is something that we are planning to explore in depth in a subsequent paper focused on *Anopheles* populations. We note that the interventions listed by the reviewer are used for *Anopheles* mosquitoes, but

not for *Aedes* mosquitoes (i.e., IRS, bednets and larviciding) and so may be more suited to a comprehensive exploration in a paper focusing on that species. That said, while there is already a lot of content in the current paper, we did consider a preliminary exploration of the detectability of fogging on *Ae. aegypti* population size and mortality rate. We have therefore added a section on "Measuring intervention impact" to the Results accordingly. We did indeed find that increases in adult mortality rate on the order of 0.05 per day were detectable with power >90%. Figure 8 has been added to depict these results.

Results: *"Following determination of optimal sampling schemes to infer adult and larval demographic parameters for Ae. aegypti populations, we explored whether CKMR methods could be used to infer the impact of an intervention on adult demographic parameters. We considered fogging with insecticides as a commonplace intervention against Ae. aegypti that is expected to increase adult mortality rate and decrease population size, and modeled this by increasing the daily lethal sampling rate by increments of 0.01 per mosquito per day, up to a maximum of an additional 0.10 per mosquito per day. Results of this analysis are depicted in Figure 8 and display a clear pattern of increasing estimated adult mortality rate, in line with that simulated, and decreasing estimated population size, reflecting the impact of increased adult mortality on the population. Figure 8 (panel C) depicts the statistical power of CKMR methods to detect a decrease in N_A or increase in mu_A as a result of this intervention given a type I error rate of 5%. The power to detect a change resulting from an adult mortality rate increased by 0.05 per mosquito per day is >90% for both N_A and mu_A, and for an adult mortality rate increased by 0.06 per mosquito per day, the power to detect a change is >99% for both parameters. This is an encouraging result for the application of CKMR methods to the assessment of intervention impact."*

"...three months, which is consistent with the length of a season, during which time the constant population size assumption in this analysis approximately holds". I think it's fine to start off, as you do here, by assuming a population at equilbrium, but I think it might be worthwhile considering how robust your inferences would be should this not be the case. One of your results which I suspect would be affected quite strongly by non-equilibrium populations is Figure 4C: which shows that sampling frequency has only minor effects on the estimates. As such, I wonder whether, *"The key result from this analysis is that CKMR estimates of NA and μA are robust for daily, biweekly, weekly, and even fortnightly collections, which is reassuring for the logistical feasibility of the method"* might be a little strong given likely variability in emergence in the field. I feel like a simulation analysis, where you allow emergence rates to vary and see when this breaks your model assumptions, would be really useful. But this may be OTT for the current manuscript, and I will leave it to the editor to determine whether they think this is in remit.

We thank the reviewer for this comment. Exploring the robustness of CKMR-based parameter inference for populations not at equilibrium is something that we plan to explore in a subsequent paper. Indeed, we do have plans to use more sophisticated population dynamic models of mosquitoes and to explore the application of CKMR inference methods to data generated from these more detailed models (the current mPlex model does not allow for seasonally fluctuating population size). We also have plans to modify the CKMR kinship probabilities and pseudo-likelihood equations so they are able to estimate a rate of population growth or decline. We see this as deserving of a more thorough analysis than would be possible in this paper, which is already quite dense. In such an analysis, substantial thought will need to go into how seasonal population fluctuations are implemented. E.g., would seasonality be modeled as a modulation in larval carrying capacity (perhaps resulting from recent rainfall), adult mortality rate (perhaps resulting from temperature variation), juvenile development rates (perhaps resulting from temperature variation), or "emergence," as suggested by the reviewer? How would parameter inference respond to varying degrees of seasonality? And how would parameter inference perform during a period of population growth, decline or some combination of both? How might the optimal sampling scheme be modified accordingly? It is also worth noting that the kinship probability equations and optimization routine are very computationally intensive, and so this has also limited the number of research questions that we have been able to explore in this paper.

In similar nature to the above, did you do any simulations when you examined how sensitive your estimates were to the number of mosquitoes found each day? I.e. if, due to clumping of captures, you captured 20 mosquitoes on some days, and zero on others. It wasn't clear to me whether you allowed stochasticity in the numbers captured each day, but I may have missed this.

Sampling is stochastic. This was not clear in the original text, as it was referred to as a sample size. In fact, there is a sampling rate rather than a size, and the rate is chosen to produce the desired size. Sampling therefore acts like any other event in the simulation (e.g., development or death) - i.e., according to a Bernoulli distribution per daily time step. The text has now been updated accordingly. The number of sampled mosquitoes therefore does vary from day to day. Regarding sensitivity to the number of mosquitoes found each day (e.g., referred to as temporal “clumping” by the reviewer), the sampling frequencies explored in panels C and D of Figure 5 explore this. Here, sampling occurs either: i) daily, ii) biweekly (in which samples are ~40 on two days of the week, and zero on the other days), iii) weekly (in which samples are ~80 on one day of the week, and zero on the other days), and iv) fortnightly (in which samples are ~160 on one day of a fortnight, and zero on the other days). Results in panels C and D of Figure 5 suggest that parameter estimates are not very sensitive to the number of mosquitoes found on each day.

"For CKMR methods, temporal information is of utmost importance, and so the day of collection should be known." As you mention, in typical surveillance programmes, this is not the case. But would you still be able to determine useful estimates should say the day of sampling be known to within 2, 3, 4, etc days? Of course, this would mean that you'd have another variable that you would need to marginalise over, but I'm not convinced it would necessarily break your analysis entirely and could substantially curb costs.

We thank the reviewer for this astute suggestion. It is true that regular sampling procedures would lead to the exact day of sampling not being known - only an interval between consecutive days of trap monitoring. Marginalizing over this variable (day of trapping) actually does not break the analysis, and as the reviewer suggests, would save a lot of money. To address this comment, we have added panels C and D to Figure 6 to explore the effect of unknown sampling day on inference of adult demographic parameters for sampling biweekly or every two days. We have also added panels G and H to Figure 7 to explore the effect of unknown sampling day on inference of larval demographic parameters, also for sampling biweekly or every two days. Updated equations are included in the SI, results are included in the relevant parts of that section, and the sentence referred to by the reviewer in this comment has also been updated in the Discussion section. The relevant sections of the Results and Discussion read as follows:

Results: *“Finally, we explored the impact that not knowing the sampling day precisely would have on estimation of N_A and mu_A. We compared cases of biweekly sampling and sampling every two days, both where the sampling day is known and where the sampling day is only known within the interval between samples (as would be the case for a regular mosquito surveillance program) (Figure 6 panels C and D). Encouragingly, we found that parameter estimation is still accurate when the precise sampling day is unknown. The precision of the parameter estimates (as measured by IQR) was not significantly different, and not knowing the precise sampling day led to slight overestimates of N_A and underestimates of mu_A; but only by a small amount - mean estimates of N_A were 3,219 and 3,206 for unknown sampling day (biweekly sampling and sampling every two days, respectively), as compared to 3,076 and 3,121 for known sampling day, and mean estimates of mu_A were 0.087 per day for unknown sampling day (for both biweekly sampling and sampling every two days), as compared to 0.091 and 0.089 per day for known sampling day (biweekly sampling and sampling every two days, respectively).”*

“Finally, we explored the impact that not knowing the sampling day precisely would have on estimation of mu_L and T_L. As for adult parameters, we compared the cases of biweekly sampling or sampling every

two days where the sampling day is known or only known within the interval between samples (Figure 7 panels G and H). Encouragingly, as for adult parameters, we found that parameter estimation was still accurate when the precise sampling day was not known. The precision of T_L estimates was unchanged (as measured by IQR) while the precision of mu_L estimates decreased slightly when the sampling day was unknown. Not knowing the precise sampling day led to slight underestimates of mu_L for biweekly sampling; but only by a small amount - the mean estimate of mu_L was 0.513 per day for unknown sampling day, as compared to 0.527 per day for known sampling day (as per Table 1, the true value is 0.554 per day).”

Discussion: *“For CKMR methods, temporal information contributes to parameter estimation, and so samples will be more informative if the day of collection is known - i.e., if samples from a mosquito trap represent collections for a single day. However, the methods still work adequately if that is not the case - i.e., if samples from a trap represent the accumulation of mosquitoes over several days, as is the case for regular mosquito surveillance programs.”*

I think it's great that the software for this paper is made openly available via: https://github.com/GilChrist19/mPlex. I did think that the CKMR repo homepage could do with a little more information on how to get the package installed, however; it would also be good to know what dependencies are required to run the simulations. Additionally, if at all possible, it would be good to have the simulations run for the manuscript included within (say) a Makefile (perhaps in a separate repository?), so that someone wanting to recapitulate the analyses would be able to straightforwardly do so. I think that something like this would be extremely helpful given the combinatorics of the experiments you perform (e.g. the variety of parameter sets covered in Figure 4), but I leave it up to the authors / editor to decide where to draw the line. Similarly, have the authors considered tagging a release of code (on Github) which was used to produce this analysis? This would be handy, especially if, in the future the codebase is modified, so that someone wanting to exactly reproduce this study could access the legacy code through the release.

We thank the reviewer for these good suggestions. We have updated the CKMR repo homepage with more information on package installation and which dependencies are required to run the simulations. This is available at: https://github.com/GilChrist19/mPlex/tree/master/CKMR. Several mPlex simulations that were run for the manuscript have been included on the MarshallLab GitHub page at: https://github.com/MarshallLab/CKMR. A release of the mPlex code used for the CKMR analyses has also been tagged and is available at: https://github.com/GilChrist19/mPlex/releases/tag/0.1.1. These changes have been annotated in the “Data availability” section of the manuscript:

“The source code for the individual-based mosquito simulation model is available at https://github.com/GilChrist19/mPlex, with a tagged version corresponding to submission of this paper available at https://github.com/GilChrist19/mPlex/releases/tag/0.1.1. Documentation, including for installation and dependencies, is available at https://github.com/GilChrist19/mPlex/tree/master/CKMR. The source code for running simulations and inferring parameters based on the pseudo-likelihood of the kinship data is available at https://github.com/MarshallLab/CKMR. Both sets of code are available under the GPL3 License and are free for other groups to modify and extend as needed.”

Is the random sampling assumption mentioned somewhere? I.e. that individuals captured in the wild represent random samples from the underlying population. I don't have as much experience with Aedes as I do with Anopheles, but I feel like this assumption might be quite crucial and could lead to biases in estimates (e.g. if host-seeking females which correlates with their age are more likely to be caught in traps). I feel like this could be touched upon in the discussion, particularly because, for Anopheles (which is proposed as an additional genus on which to use this method), it is likely an issue.

We have added discussion of the random sampling assumption in the Discussion section, according to the reviewer's advice, as it applies to both Aedes and Anopheles mosquitoes. We have included mention of potential biases towards sampling adult females of a certain age, and related to spatial clustering of samples, which could artificially elevate the number of related pairs found. The additional text follows:

"An underlying assumption of CKMR analyses is random sampling, which should be noted throughout field protocols. E.g., spatial clustering of samples should be avoided as this can artificially elevate the number of pairs found, and biases towards trapping mosquitoes of certain ages (e.g., those more likely to host-seek) should be avoided where possible, and accounted for where not."

Minor comments:

Throughout the manuscript, the word *"consistent"* appears (49 times!). For instance, *"considering all consistent event histories"*, *"...for consistency with the larva being present on the day of sampling..."*, *"Adult offspring at time t2 from all adult females at consistent times"*. I think in quite a few of the language could be simplified, often because the quantities are at equilibrium, so consistency isn't really needed. I also didn't necessarily find the use of *"consistent"* to be that illuminating: e.g. *"is summed over days (0 − TE − TL − TP − TA) through (0 − TE − TL − TP), for consistency with the adult offspring being present on the day of sampling " could be changed to "is summed over ... through ... to allow for the range of conceivable times when the adult sampled on day 0 was born as an egg."*

We have reduced the number of times that the word *"consistent"* is used (now 9). We have also reduced the number of times that the word *"consistency"* is used. Where their use remains, we do feel that these word choices are appropriate, as they describe the timing of events that are consistent with mosquito life history, as modeled in the paper.

I understand eq. (8) and why p_a(t) -- the probability that a given adult in the population has age t -- enters the equation in the way it does. But it took me a bit of time to understand it. I think it would be good to give a little more explanation of this. I think part of the confusion is the sentence, *"pA(t1 − ti) represents the probability that the mating event occurs on day (t1 − ti)."* -- it does, but really this just represents the probability that a mosquito is of age (t1-ti).

We thank the reviewer for this observation. They are correct that pA(t1-ti) represents the probability that the mosquito is of age (t1-ti), and equivalently that the mating event occurs on day ti (as opposed to day (t1-ti)). We have now added language to clarify this throughout the manuscript, including the description of the kinship probabilities for father-offspring, full-sibling and half-sibling pairs.

I agree with assuming that the adult (and other life stages') mortality rate is independent of age, but I think it's worth saying this somewhere.

Thank you for this suggestion. We have added mention of mortality rates being independent of age in Materials & Methods section 2.1: Mosquito population dynamics. We have also mentioned relaxing this assumption as an area for future exploration in the Discussion section.

The panels in Figure 2 are not referred to in order: panel B is referred to first, for instance. I think it would be nice if the panels followed the order of the text, since the reader then doesn't find themselves thinking, "Did I miss something?"

Thank you for this suggestion. We have reordered the panels of Figure 2 so that they now match the order of mention in the text.

Why not put a legend with *"circle -> living individuals; square -> sampled individuals"* in figure 2 somewhere as there's quite a bit of space. Also, what do the colours mean? E.g. grey in panel E.

Thank you for this suggestion. We have now added a legend explaining the shapes and colors in Figures 2 and 3.

"For convenience, let us refer to the larva sampled on day t1 as individual 1" -- how about *"cohort 1"* -- I found the *"individual"* in this confusing since there are many.

This is correct as-is. The sibling kinship probabilities are calculated based on a single reference individual sampled on day t1.

"pL(t1 − y1 − TE) represents the probability that egg 1 is laid on day (t1 − y1 − TE)". I think it would be more useful to introduce what p_L(t) is (i.e. the probability that a given larva has a given age) first. Then pL(t1 − y1 − TE) is just the probability that a larva is of age (t1 − y1 − TE).

We thank the reviewer for this observation, which is equivalent to the earlier observation of how we had described equations having pA(t). They are correct that pL(t1−y1−TE) represents the probability that the larva is of age (t1−y1−TE), and equivalently that the day of egg-laying is y1 (as opposed to day (t1−y1−TE)). We have now added language to clarify this throughout the manuscript, including the description of the kinship probabilities for full-sibling and half-sibling pairs.

"the size of each sampling event is sufficiently low relative to the total population size" -> *"each sample taken is small relative to the overall population"*

Thank you. We have made this change.

"The kinship probabilities for each pair of individuals are assumed to be independent of each other, even though they are not." I need a little hand-holding here: can you perhaps add a sentence or so that explains this in more detail? Is this just saying that, by removing a small number of individuals from the population, when sampling, does not change the underlying distributions by much? So, for example, in equation (22) you assume that finding one individual with a given mother does not affect your probability of finding another. In other words, you assume that you sample with replacement whereas, in reality, this is not the case.

We rewritten this passage to clarify ambiguity over why the pseudo-likelihood approach is needed. The rewritten passage now reads: *"Here, we combine the probabilities of parent-offspring and sibling pairs in a manner that takes advantage of the nature of the kinship probabilities and the sampling process. The joint kinship distribution is too complicated to be expressed analytically as a full likelihood, and so we instead adopt a "pseudo-likelihood" approach [1], which treats the marginal kinship probabilities for each pair of sampled individuals separately."* The pseudo-likeilhood approach is not necessitated by sampling without replacement - it is also required where sampling were to be carried out with replacement. It is instead an issue of the dependence of some kinship relationships on eachother, and the full likelihood needing to address these jointly. E.g., if A is a sibling of B and B is a sibling of C, then A is also a sibling of C. The pseudo-likelihood approach ignores the kinship relationships between A-and-B and B-and-C; however, under sparse sampling, the expected marginal information from a comparison is similar to its expected conditional information given all other comparisons, so approximate independence is reasonable.

I feel that writing equation (22) (and other similar equations, e.g. eq. (25)) as: k ~ binomial(n_A, p_MOA) or binomial(k|N_A, p_MOA) rather than writing out the explicit binomial likelihood would be more illuminative and also condense things. Then equation (23) could be written as a sum over

binomial_log(k|N_A, p_MOA) which would mean the reader has to do less work to follow subscripts and indices.

We thank the reviewer for this excellent suggestion. We have updated Equations 17, 18, 20 and 21 accordingly.

"Additionally, when counting siblings, we only consider siblings with indices > i to avoid double-counting." I am not sure where this enters equation (25) or (26)?

Thank you. It applies to both equations, and we have now moved the sentence so that it precedes the first equation to clarify this. Application to the second equation follows in the sense that the second is a summation over the first.

"Functionality is included to account for spatial population structure, with mosquitoes being distributed across populations in a metapopulation [26], and each population having an equilibrium adult population size, N∗ A, and exchanging migrants with the other populations; however, in the present analysis, a single panmictic population is modeled." Given that spatial modelling is not included in this analysis, I am not sure it needs to be mentioned as an inattentive reader might start thinking that the model used is spatial?

Thank you. We have removed the description of the spatial features of the simulation model.

"Daily mortality rates are as defined earlier and implemented according to a Bernoulli distribution for each individual." -> *"Individual survival through a given day is assumed to follow a Bernoulli distribution with a stage-appropriate survival probability."*

Thank you. We have made this change.

"Sex is modeled at the adult stage - half of pupae emerge as females, and the other half as males, implemented according to a Bernoulli distribution with probability 0.5" -> *"The sex of an emergent pupa is drawn from a Bernoulli distribution with probability 0.5, so that, on average, half adults are female and half male."*

Thank you. We have made this change.

"logistically-feasible" -> *"logistically feasible"* since words ending in *"ly"* typically don't need hyphens

Thank you. We have made this change throughout the manuscript.

"by requiring integration over six latent event times" - I know what you mean but, since your analysis is all discrete, you might want to change *"integration"* to *"summing"*?

We thank the reviewer for pointing this out. This change has been made throughout the manuscript.

"To estimate adult parameters, our default sampling scheme consisted of a total of 1,000 sequenced individuals sampled daily..." -- sounds like a lot of mosquitoes until I read the bit in parentheses. It might sound less alarming if you said, *"To estimate adult parameters, our default sampling scheme consisted of a 3 month window when, overall, 1000 mosquitoes were sampled, with roughly uniform numbers of mosquitoes sampled each day (i.e. 11-12 mosquitoes per day)."*

We thank the reviewer for this suggestion. The total sample size of 1,000 sequenced individuals does sound daunting unless immediately clarified. We have edited the new text accordingly to align with the

reviewer's advice: *"To estimate adult parameters, our default sampling scheme consisted of a total of 1,000 sequenced individuals sampled daily over a three-month period (i.e., ca. 11-12 individuals sampled each day, for a total of 1,000 individuals after three months of sampling)."*

"limited to scenarios where the number of sampled adult females was greater than or equal to the number of sampled adult males" -- I spent a while looking for this ratio until I interpreted what your labels meant in Fig. 4A (e.g. 50F25M25L). I feel that putting a short sentence to explain one / a couple of these would help the slow reader like myself.

We thank the reviewer for this good suggestion. We have added a clarification to the figure legend according to their advice: *"...limited to scenarios where the number of sampled adult females exceeds the number of sampled adult males (e.g., "75F25M" represents a sample consisting of 75% adult females and 25% adult males, and "50F25M25L represents a sample consisting of 50% adult females, 25% adult males, and 25% larvae")."*

Out of interest, what do you think leads to the bias seen in Figures E and F for short durations of sampling?

This is at least in part due to the elevated mortality rate and suppressed population size resulting from the same sample size (1,000) being obtained by lethal sampling in a shorter time frame. We have edited the text to reflect this: *"These results suggest that sampling durations of 3-4 months provide unbiased estimates of N_A and mu_A, while sampling durations of 1-2 months lead to significantly higher estimates of mu_A and lower estimates of N_A, at least partly due to the elevated mortality rate and suppressed population size resulting from the same amount of lethal sampling being compressed into a shorter time frame."*

"This confirms that the parameter estimates from the optimal sampling scheme in Figure 4 are indeed optimal..." -- I'm not sure I understand why this follows from the previous sentence.

This has been clarified in the edited version of this text: *"Interestingly, adult parameter estimates inferred from combined parent-offspring and full-sibling pseudo-likelihood components are more accurate and precise (as measured by the median and IQR of replicate parameter estimates, respectively) compared to those inferred from either pseudo-likelihood component in isolation (Figure 5). This confirms the validity of the optimal sampling scheme inferred in Figure 4, which was inferred for combined parent-offspring and full-sibling pseudo-likelihood components."*

"Curiously, we found that including half-siblings in our analyses introduced significant biases in our parameter estimates, leading to an underestimate of μA and an overestimate of NA" I also found this quite strange and I wonder whether there are any ways to probe this further?"

We agree with the reviewer that it would be valuable to probe this further. Unfortunately, the run-time for CKMR analyses including half-siblings is several weeks to a month, due to the large number of latent event times that need to be summed over. We therefore chose to table probing this further; but plan to revisit once we have developed more efficient approaches to CKMR pseudo-likelihood calculation.

"spatially-structured" -> " spatially structured"

Thank you. We have made this change throughout the manuscript.

Reviewer #2:

Comments:

This is a great paper. It took me longer than I expected to review it, because the analyses are quite involved, but this is unavoidable (both the complexity and the delay). The importance of the question justifies the intensity of the analyses. I support its publication in its current form because I think the analyses are novel and sophisticated, and because the application is internationally important and impactful.

We thank the reviewer very much for their interest in and support of our work.

I've got one major criticism of the work, and one minor. The central problem is acknowledged by the authors – that it's pretty easy to fit a model to matching synthetic data. To my mind, model structural error is a big deal – almost big enough to cast doubt on all the results shown here. The problem is that there's no solution to the problem. All you can do is make the case quite emphatically. Thus, I'd recommend that the authors spend a few more sentences talking about this issue.

We thank the reviewer for their suggestion and agree that model structural error is a big issue. This is something that we plan to explore in subsequent papers. For now, per the reviewer's suggestion, we have added a few sentences to the paragraph of the Discussion that addresses this issue. We include the relevant paragraph here for ease of reference:

"As a preliminary exploration of the application of CKMR methods to mosquitoes, and as a modeling exercise, this study has several limitations. Firstly, the same life history model (Figure 1) was used as a basis for both the population simulations and the CKMR analysis. Additionally, other than the parameters being estimated, the same parameters were used in both simulations and analysis. This represents an overly generous scenario as compared to the field, where true life history is varied and complex, and where life history parameters are only approximately known. That said; this is an appropriate starting point to verify the utility of the method for mosquitoes - it first needs to be shown to infer the true value of a parameter given the true model. Subsequent analyses should explore the robustness of parameter inference when other parameters in the model are dynamic or misspecified, or when kinship data are generated from a more detailed model. The CIMSiM model of Ae. aegypti population dynamics [22], for instance, models juvenile dynamics at the container level and incorporates temperature-driven development rates. Using CKMR methods on data from a detailed model like CIMSiM would allow the impact of structural model differences to be explored to a degree, while acknowledging that true ecological dynamics are more complex than those of any in silico model. The impact of modest model variations could also be explored, such as age-dependent mortality rates and increased variance in the fecundity parameter, beta. Presently, the daily number of offspring generated by each adult female is Poisson-distributed and distributing this according to an overdispersed negative Binomial distribution would reduce the effective population size, N_e, while maintaining the census adult population size, N_A [13], the impact of which would be interesting to explore."

My second, and more minor problem, is the reliance on comparisons with marine fish studies. This might be a problem, given how different fish and mosquitoes are. It's fine to adopt methods from different contexts - dispersal is dispersal, and kinship is kinship. However, type 1 and 2 error rates resulting from the application and analysis of SNP may differ. I'd be really careful what you extrapolate from one context to this one.

We thank the reviewer for their comment. We believe our paper represents more of an "adaptation" of methods rather than an "adoption.". The methods used for fish have been adapted to an egg-larva-pupa-adult life history for mosquitoes, as well as a mating structure in which females tend to mate once upon emergence, while males may mate more than once and throughout their adult lifespan. Regarding tolerable type 1 and 2 error rates for kinship inference, exploring this in further detail is beyond the scope of this initial paper, as we have assumed perfect kinship inference here (as have other theoretical papers

in the CKMR field). The accuracy of kinship inference is certainly something that we will explore further in subsequent papers, in particular, considering that we have scheduled mosquito field work and sequencing for later this year. However, we do expect the lessons from fish studies to broadly apply here - type 1 errors (false positive kinship pairs) intuitively will impact parameter inference more than type 2 errors (false negative kinship pairs), and as mentioned in our paper, *"Kinship inference methods should be calibrated accordingly."* Trading off between type 1 and 2 error rates is something that should be achievable for mosquito species; but we will explore this in earnest in a paper describing the results of our field work.

References:
****

- Hillary RM, Bravington MV, Patterson TA, Grewe P, Bradford R, Feutry P, Gunasekera R, Peddemors V, Werry J, Francis MP, Duffy CAJ, Bruce BD (2016) Genetic relatedness reveals total population size of white sharks in eastern Australia and New Zealand. Scientific Reports 8: 2661.
