# Supplementary material for: Close-kin mark-recapture methods to estimate demographic parameters of mosquitoes
Source: PLoS Comput Biol. 2022 Dec 12;18(12):e1010755. doi: 10.1371/journal.pcbi.1010755 (PMC9779664; doi:10.1371/journal.pcbi.1010755)
Supplement: Attachment — Submitted filename: sharma2022_second_review_round.pdf [file pcbi.1010755.s004.pdf]

I thank the authors for their comprehensive response to my comments, which has answered the majority of my queries. The below are further minor comments. Should these be addressed, I am happy for the paper to be published in this journal.

I really like the new, fully annotated, Figures 2 and 3. It makes it much easier to understand the calculations.

I really like Figure 4, but I confess I do not fully understand its implications. What is the intuition behind why the mother is more likely to be sampled *after* the larva whereas fathers are always more likely to be sampled *before* their offspring? I think a sentence or so explaining this intuition would really help readers (like me!) to understand.

A minor point but the bottom axis on Figure 4 is a bit misaligned (specifically the t_2 and t_1).

I didn't realise on my initial reading that the mosquito model that you simulated from assumed deterministic transitions between life stages. (This was an omission on my part.) I think this is fine, but I think it worth considering stochastic transitions in further work: I realise this would increase the cost of calculating the probabilities, but it would be interesting as an additional piece of work to consider whether assuming deterministic life stage values breaks inference when these are random (my guess is that it wouldn't break things, it would just make it harder to infer parameters). As such, I would consider adding something to this effect to the discussion: you already discuss model misspecification, but I still think it worth including something explicit about stochastic vs deterministic life stages.

A minor point, but the apostrophe in Figure 5 caption is not quite right: "75F25M" (the first one is the wrong way around) and same for other similar cases in the caption.

In the caption of Figure 5, there is the statement: "The optimal sampling scheme consists of a total of 1,000 adult females collected biweekly-to-fortnightly over a three month period." I would remove this since this is the only place in the caption where you discuss a result (which is already covered in the text and without the accompanying reasoning regarding killing adults, it may confuse).

I'm very pleased that the results hold up when the exact sampling day of a specimen is unknown: that's great for programatic rollout of this method. I realise that you have amended the supplementary materials to reflect this marginalisation, but in the main text, this isn't (as best I could see) referred to. A result of this is that it is not clear whether: a) you used the same analysis as you did in other parts, assuming that the samples were (say) all collected in the middle of the interval or b) whether you marginalised as I think you did. I would suggest having a short subsection in the methods where you briefly discuss how you did this (referring to supp mat for involved calculation), since I think this addresses a really important practical concern of this method.

On the same note, is there a reason that in Fig 6C / D you did not consider weekly collections (both known and unknown)? Again, in my experience, weekly collections are quite common, and, if this sufficed, then great.

In Figure 6 caption, I would remove "Estimates of NA and μA are accurate regardless of whether the sampling day is precisely known" since again this is repetitious of the text, where the results are discussed in more detail.

Can I check, does "The precision of the parameter estimates (as measured by IQR) was not significantly different" mean "significance" in the context of statistical testing or in the context of not representing a meaningful difference? If the latter, I would change "significantly" to "substantively" or something without this ambiguity. (And similarly so for other instances of this word.)

I like the new analysis that underpins figure 8. Can you add the true values of N and mu to plots A and B (as perhaps a dashed line)?

I would remove "The statistical power to detect this is > 90% for fogging-induced mortality rates of 0.05 per mosquito per day or higher." from the caption in Figure 8 since this replicates the text.

Can I suggest that you include info about the statistical test you used to determine a difference in N or mu? (I presume you did a LR test / score test or something?) Also, how many replicates were used to determine the power?

I noticed that "Binomial" is capitalised in the discussion (it may be elsewhere too); it shouldn't be since this is not a name (versus say "Poisson").

Lastly, the changes to mPLEX Github repo look great. I would say that the *https://github.com/MarshallLab/CKMR* repo could do with a README which explains what the files are (you may also want to consider putting things into folders to make it easier to understand the purpose of different files).
