# Supplementary material for: Close-kin mark-recapture methods to estimate demographic parameters of mosquitoes
Source: PLoS Comput Biol. 2022 Dec 12;18(12):e1010755. doi: 10.1371/journal.pcbi.1010755 (PMC9779664; doi:10.1371/journal.pcbi.1010755)
Supplement: Attachment — Submitted filename: SharmaEtAl_2022_R2_ResponseToReviwers.pdf [file pcbi.1010755.s005.pdf]

Berkeley Public Health

UNIVERSITY OF CALIFORNIA, BERKELEY

DIVISIONS OF BIOSTATISTICS & EPIDEMIOLOGY
SCHOOL OF PUBLIC HEALTH
2121 BERKELEY WAY #5302
BERKELEY, CA 94720

29 October 2022

To: Drs. Penny Hancock and Ville Mustonen, PLoS Computational Biology

Re: PCOMPBIOL-D-22-00257R1

Please find enclosed our revised manuscript entitled, “Close-kin mark-recapture methods to estimate demographic parameters of mosquitoes” for consideration as a research article in PLoS Computational Biology. We greatly appreciate both rounds of reviewers’ comments and believe we now have a greatly refined manuscript thanks to their thoughtful comments. We have constructively addressed each point below, with reviewer comments in black text, our response in blue text, and quotations from the revised manuscript in blue italics text and quotation marks. We thank you and the two reviewers very much for your time and for improving the quality of our manuscript. We look forward to hearing your response regarding the revised manuscript.

Yours sincerely,

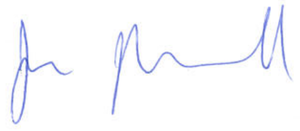

Dr John Marshall, on behalf of the authors

Associate Professor in Residence
Divisions of Epidemiology & Biostatistics
School of Public Health
University of California, Berkeley
Phone: +1-510-664-4724
Email: john.marshall@berkeley.edu
Website: www.MarshallLab.com

Reviewer comments:
****

Editors:

Comments:

Thank you very much for submitting your manuscript “Close-kin mark-recapture methods to estimate demographic parameters of mosquitoes” for consideration at PLOS Computational Biology. As with all papers reviewed by the journal, your manuscript was reviewed by members of the editorial board and by several independent reviewers. The reviewers appreciated the attention to an important topic. Based on the reviews, we are likely to accept this manuscript for publication, providing that you modify the manuscript according to the review recommendations. The reviewers appreciate that most of their suggestions have been addressed. Reviewer 1 has made further suggestions to improve the readability and clarity of the manuscript.

Thanks very much for your feedback, and for taking the time to carefully review our work. We are happy to hear that the manuscript is likely acceptable for publication. We appreciate Reviewer 1's constructive suggestions to improve the manuscript, and address these below.

Reviewer #1:

Minor comments:

I thank the authors for their comprehensive response to my comments, which has answered the majority of my queries. The below are further minor comments. Should these be addressed, I am happy for the paper to be published in this journal.

Thank you for your careful reading and thoughtful comments. We believe they have improved the manuscript greatly.

I really like the new, fully annotated, Figures 2 and 3. It makes it much easier to understand the calculations.

Thank you for requesting we improve these two figures. We agree that they provide a nice way to explain the kinship probability calculations.

I really like Figure 4, but I confess I do not fully understand its implications. What is the intuition behind why the mother is more likely to be sampled after the larva whereas fathers are always more likely to be sampled before their offspring? I think a sentence or so explaining this intuition would really help readers (like me!) to understand.

This is best understood with reference to the schematic diagrams in Figures 2 and 3. We have added the following text and figure references to describe this:

"For mother-larval offspring pairs, for instance, the mother is more likely to be sampled after the larval offspring since, after egg-laying, the mean time to maternal sampling is longer than the duration of the egg stage plus the mean time to larval sampling (Figure 2, panel B). Conversely, for father-adult offspring pairs, the father is more likely to be sampled before the adult offspring since, after mating, the mean time to paternal sampling is shorter than the time to maternal egg-laying combined with the duration of offspring juvenile development plus the mean time to adult offspring sampling (Figure 2, panel D). Siblings are most likely to be sampled around the same time as each other, while half-siblings may be found the largest number of days apart (Figure 3)."

A minor point but the bottom axis on Figure 4 is a bit misaligned (specifically the t_2 and t_1).

This has been corrected.

I didn't realise on my initial reading that the mosquito model that you simulated from assumed deterministic transitions between life stages. (This was an omission on my part.) I think this is fine, but I think it worth considering stochastic transitions in further work: I realise this would increase the cost of calculating the probabilities, but it would be interesting as an additional piece of work to consider whether assuming deterministic life stage values breaks inference when these are random (my guess is that it wouldn't break things, it would just make it harder to infer parameters). As such, I would consider adding something to this effect to the discussion: you already discuss model misspecification, but I still think it worth including something explicit about stochastic vs deterministic life stages.

The following text has been added to the Discussion section: *"The impact of modest model variations could also be explored, such as… variance in the duration of juvenile life stages…"* Currently, the mortality rates of juvenile life stages are stochastic; but durations of the egg, larval and pupal stages are constant. Introducing variance into these durations would allow the stochasticity that the reviewer refers to to be explored.

A minor point, but the apostrophe in Figure 5 caption is not quite right: "75F25M" (the first one is the wrong way around) and same for other similar cases in the caption.

This has been corrected.

In the caption of Figure 5, there is the statement: "The optimal sampling scheme consists of a total of 1,000 adult females collected biweekly-to-fortnightly over a three month period." I would remove this since this is the only place in the caption where you discuss a result (which is already covered in the text and without the accompanying reasoning regarding killing adults, it may confuse).

The suggested change has been made.

I'm very pleased that the results hold up when the exact sampling day of a specimen is unknown: that's great for programatic rollout of this method. I realise that you have amended the supplementary materials to reflect this marginalisation, but in the main text, this isn't (as best I could see) referred to. A result of this is that it is not clear whether: a) you used the same analysis as you did in other parts, assuming that the samples were (say) all collected in the middle of the interval or b) whether you marginalised as I think you did. I would suggest having a short subsection in the methods where you briefly discuss how you did this (referring to supp mat for involved calculation), since I think this addresses a really important practical concern of this method.

Thank you for this suggestion. We had updated the code on GitHub, but not the manuscript or S1 Text, to address cases of unknown sampling day. We were able to describe how we marginalized over sampling day in a single equation (Equation 16), and so have now included this in the Methods section of the revised text (Section 2.2.6).

On the same note, is there a reason that in Fig 6C / D you did not consider weekly collections (both known and unknown)? Again, in my experience, weekly collections are quite common, and, if this sufficed, then great.

As stated in the text, *"We decided to focus on biweekly sampling… given its precedent in the field, and considering it allows more mosquitoes to be collected than weekly sampling,"* and, *"We explored whether only knowing the sampling day to an accuracy of 2-4 days (the number of days between collections) would substantively impact parameter inference."* Biweekly sampling was favored given the large sample sizes required for CKMR analyses.

In Figure 6 caption, I would remove "Estimates of NA and μA are accurate regardless of whether the sampling day is precisely known" since again this is repetitious of the text, where the results are discussed in more detail.

The suggested change has been made to both the Figure 6 and Figure 7 caption.

Can I check, does "The precision of the parameter estimates (as measured by IQR) was not significantly different" mean "significance" in the context of statistical testing or in the context of not representing a meaningful difference? If the latter, I would change "significantly" to "substantively" or something without this ambiguity. (And similarly so for other instances of this word.)

The reviewer is correct that this refers to a meaningful difference. We agree with the reviewer's suggestion and have replaced "significantly" with "substantively" in three instances.

I like the new analysis that underpins figure 8. Can you add the true values of N and mu to plots A and B (as perhaps a dashed line)?

The suggested change has been made for adult mortality rates (i.e., the intervention-free mortality rate plus that induced by fogging). Adding the true population size is a bit complicated, as population size is dynamic through the course of the sampling/intervention, particularly for larger fogging-induced mortality rates for which the population is being cumulatively suppressed over time. The correspondence between estimated and actual population size is something we would like to investigate further in a subsequent study on estimating intervention impact.

I would remove "The statistical power to detect this is > 90% for fogging-induced mortality rates of 0.05 per mosquito per day or higher." from the caption in Figure 8 since this replicates the text.

The suggested change has been made.

Can I suggest that you include info about the statistical test you used to determine a difference in N or mu? (I presume you did a LR test / score test or something?) Also, how many replicates were used to determine the power?

The following text has been added to address this comment: *"Here, a one-tailed test was used given a type I error rate of 5% and 100 simulation-and-analysis replicates for each case."*

I noticed that "Binomial" is capitalised in the discussion (it may be elsewhere too); it shouldn't be since this is not a name (versus say "Poisson").

This has been corrected.

Lastly, the changes to mPLEX Github repo look great. I would say that the https://github.com/MarshallLab/CKMR repo could do with a README which explains what the files are (you may also want to consider putting things into folders to make it easier to understand the purpose of different files).

Thank you for these suggestions regarding the MarshallLab CKMR GitHub repo. We have added a README to the repo, and have put files into folders, as suggested.
